# Integrating multimodal data to predict the progression of hormone-sensitive prostate cancer

**DOI:** 10.1186/s12014-025-09543-7

**Published:** 2025-05-29

**Authors:** Xiangfu Lu, Chenxi Pan, Luhan Yao, Jiayu Wan, Xiaolong Xu, Wei Wang, Xiangying Wang, Xiaoyun Liu, Zhonghua Jin, Hongyu Wang, Yi He, Bo Yang

**Affiliations:** 1 Department of Urology, 967 th hospital of PLA Joint Logistics Support Force, No.80 Shengli Road, Dalian, 116014 PR China; 2 State key laboratory of fine chemicals, Frontiers Science Center for Smart Materials Oriented Chemical Engineering, School of Bioengineering, Dalian University of Technology, Dalian, 116023 PR China; 3 School of Information and Communication Engineering, Dalian University of Technology, Dalian, Dalian, 116023 PR China; 4 Department of Urology, The Second Hospital of Dalian Medical University, No.467 Zhongshan Road, Dalian, 116023 PR China; 5 Department of chest surgery, The Second Hospital of Dalian Medical University, No.467 Zhongshan Road, Dalian, 116023 PR China; 6 The Second Hospital of Dalian Medical University, 467 Zhongshan Road, Dalian, 116023 Liaoning China

**Keywords:** Hormone-sensitive prostate cancer, Histopathology, Proteome, Magnetic resonance imaging, Machine learning

## Abstract

Identifying the population at risk of rapid progression from hormone-sensitive prostate cancer (HSPC) to lethal castration-resistant prostate cancer (CRPC) is a challenge. This work has highlighted important prognostic insights based on proteomics data, magnetic resonance imaging (MRI) and histopathological specimens. We retrospectively developed a multi-omics-based model based on 77 patients with HSPC. In order to identify the features related to survival time under each mode, we used the Boruta algorithm for feature screening. In order to demonstrate the effectiveness of our selected features, we used six machine learning methods to validate the classification of the selected features for each mode. A total of 63 proteome signatures, 60 HE signatures, 56 T2WI signatures, and 54 ADC signatures were identified as features related to the speed of HSPC progression. Ultimately, 30 multi-omics-based features were determined by the least absolute shrinkage and selection operator (LASSO) method and multivariate cox regression. In order to stratify patients with significant disparities in progress, a nomogram model was developed, of which the C-index was 0.906. Accordingly, the developed model could help identify patients who are at a high risk of rapid CRPC progression, and aid clinicians in guiding personalized clinical management and decision-making.

## Introduction

Globally, prostate cancer (PCa) is the fifth most common cause of cancer-related mortality and the second most common cancer in men [1]. For individuals with locally advanced and metastatic prostate cancer, androgen deprivation therapy (ADT) in association with androgen blocking is frequently effective during the early phases of treatment [2]. Only 5–10% of patients survive 10 years after starting ADT, and nearly all hormone-sensitive prostate cancers (HSPC) develop into castration-resistant prostate cancers (CRPC) within five years [3]. PCa has a complicated illness spectrum with subtypes ranging from clinically indolent to aggressive due to its heterogeneity. Patients’ progress spans to CRPC differ greatly from one another, although little research has been done to investigate this. The theory by Hussain et al. states that if the CRPC stage developed within the first seven months of ADT, the probability of death would increase fourfold [4]. Several randomized controlled phase-III trials, such as CHAARTED and LATITUDE [5, 6], have shown that early and appropriate follow-up strategies can be implemented to optimize treatment regimens in HSPC patients who are found to have either a short- or long-term progression to CRPC prior to treatment initiation.

Radiomics combined with machine learning (ML) techniques have been used to differentiate between low- and high-grade PCa [7–11], provide tumor description [12–14], conduct risk assessment [15, 16] and plan treatment strategies [17, 18]. HSPC studies based on radiomics are unavailable. However, MRI has been valuable in clinical monitoring and therapy of metastatic HSPC [19]. Therefore, the in-depth analysis of HSPC imaging data may play a significant role in the precision treatment of HSPC.

At the microscopic scale, the routine HE staining of tissue biopsies enables pathological diagnosis prior to therapeutic intervention. Clinically, the diagnosis of pathology is strongly dependent on the clinician’s experience, and the specific pathological diagnosis is very time-consuming. When the diagnosis cannot be confirmed via HE biopsy, immunohistochemistry is utilised. However, immunohistochemical analysis tedious and time-consuming. It delays the patient’s clinical diagnosis and treatment decision making. Currently, Gleason-sum score is primarily used to evaluate the stage of PCa. In previous studies, we found that the Gleason-sum score in disease progression had a strong limitation [20]. However, the clinical understanding of Pathomics is limited currently, so the use of omics in pathology for the clinical study of HSPC is imperative. In other cancer types, studies of whole-slide images (WSIs) have advanced our ability to quantify the histopathological architecture of tumours using deep learning [21, 22] and interpretable features [23, 24]. The deep learning of WSIs may contribute to quantifying the rate of disease progression in patients with HSPC.

Proteomics, reflecting cellular responses to genomic, epigenomic, and environmental alterations, could be used to identify the molecular mechanisms underlying HSPC progression to CRPC, which was confirmed in our previous work [20]. Conceptually, Integrating proteomics data with other forms of data from genomes, image-omics, and phenomics creates a more comprehensive picture, which has the potential to unveil novel biology and improve clinical practice. Realizing this promise necessitates the development of computational methodologies and tools capable of driving efficient multi-omics data integration. In clinical, multi-scale clinical imaging is always routinely performed during the course of care, including magnetic resonance imaging (MRI) at a mesoscopic scale and hematoxylin and eosin (HE)-stained slides at a microscopic level. We thus hypothesise that multi-scale imaging incorporates complementary information, rather than merely recapitulating proteomic insights of prognostic value [25]. To fully utilize this potential, computational techniques and instruments capable of facilitating efficient multi-omics data integration are needed.

In this work, we analysed the complementary prognostic and multi-modal features derived from proteomic, histopathological and radiological data from patients diagnosed with HSPC. Accordingly, we have collected multi-omics data from patients with HSPC to develop a predictive model of HSPC progression and verify its accuracy. Our results reveal the empirical advantages of cross-modal integration and demonstrate the ability of multi-modal machine-learning models to predict the rates of HSPC progression.

## Materials and methods

### Patients

This retrospective study was approved by the ethics committee of the Second Hospital of Dalian Medical University and is compliant with the principles of the Declaration of Helsinki (approval number: 2023064). All the patients signed written informed consent before participation. The patient cohort, including 77 locally advanced or metastatic HSPC was the same across our proteomics work, except for one due to missing radiological data [20]. The clinical characteristics are listed in Table 1.

**Table 1:** Representativeness of study participants

	Clinical characteristics	Number
Age	Mean ± SD	72.77 ± 8.66
	Median [min-max]	74.00 [50.00,89.00]
Gleason	5 + 5	4 (5.19%)
	5 + 4	16 (20.78%)
	5 + 3	3 (3.90%)
	4 + 5	12 (15.58%)
	4 + 4	19 (24.68%)
	4 + 3	14 (18.18%)
	3 + 5	5 (6.49%)
	3 + 4	4 (5.19%)
T-stage	T4	40 (51.95%)
	T3	24 (31.17%)
	T2	13 (16.88%)
N-stage	N1	53 (68.83%)
	N0	24 (31.17%)
M-stage	M1c	9 (11.69%)
	M1a/b	61 (79.22%)
	M0	7 (9.09%)
GleasonSUM	9	28 (36.36%)
	8	27 (35.06%)
	7	18 (23.38%)
	10	4 (5.19%)
t-PSA	< 50	11 (14.29%)
	50–100	14 (18.18%)
	> 100	52 (67.53%)
Time to CRPC (months)	Mean ± SD	14.70 ± 14.00
	Median [min-max]	9.00 [2.00,65.00]

## Proteomics sample Preparation and data analysis

The pathologists evaluated the primary Gleason patterns and detailed the boundaries of the cancerous changes on the FFPE. The pre-ADT samples were extracted (diameter 1 mm) from the FFPE blocks at the histopathological sites, thus ensuring that all samples were derived from cancerous tissue. A total of 78 tissue core samples were collected and then assigned to two groups: a discovery set (*n* = 16) and a modeling set (*n* = 62). These samples were then analyzed using the PCT-PulseDIA technology.

The PCT-assisted proteomics sample preparation procedures followed our previously published workflows [14]. In brief, about 0.2 mg of FFPE punches were dewaxed with heptane, hydrated with ethanol, and then underwent acidic hydrolysis by 0.1% formic acid (FA, Thermo Fisher Scientific, USA) and basic hydrolysis by 0.1 M Tris-HCl (pH = 10.0). Samples were next lysed using a 6 M urea/2 M thiourea buffer (Sigma, USA), reduced by tris (2 carboxyethyl) phosphine (Sigma, USA), and alkylated by iodoacetamide (Sigma, USA). The lysates were then digested using PCT by a mix of Lys-C and trypsin (Hualishi Tech. Ltd., China). Finally, the PCT-assisted digestion reaction was stopped by trifluoroacetic acid and cleaned by C18.

A total of 400 ng peptides were injected and separated along a 45 min liquid chromatography gradient (from 3 to 28% buffer B– see below for its composition) at a flow rate of 300 nL/min (precolumn: 3 μm, 100 Å, 20 mm × 75 μm i.d.; analytical column: 1.9 μm, 120 Å, 150 mm × 75 μm i.d.). Buffer A was mass spectrometry-grade water containing 2% acetonitrile and 0.1% FA; buffer B was acetonitrile containing 2% H_2_O and 0.1% FA. The peptides were then analyzed by a Q Exactive HF hybrid Quadrupole-Orbitrap (Thermo Fisher Scientific, USA) using the PulseDIA mode with four pulses, as previously described [15].

To analyze the PulseDIA data, we generated an experimental spectral library for the PCa tissues. We combined the cleaned peptides from the discovery dataset into a mixture containing 100 µg peptides. The peptide pool was then separated using Thermo Ultimate Dinex 3000 (Thermo Fisher Scientific, USA) with an XBridge Peptide BEH C18 column (300 Å, 5 μm x 4.6 mm x 250 mm) (Waters, Milford, MA, USA) and a 60 min gradient. Finally, we collected 20 peptide fractions. The fraction data were acquired using data-dependent acquisition (DDA). Four fractions were randomly selected and analyzed by MS twice. Spectronaut™ Pulsar X (version 14.6, Biognosys, Switzerland) was used to generate the spectral library [16]. The DDA files were searched by Pulsar against a human Swiss-Prot FASTA database (downloaded on 2020-01−22), including 20,367 protein sequences; the settings were left to their default values. The established library comprised 143,347 peptide precursors, 115,257 modified peptides, and 9,644 proteins. Next, PulseDIA files were analyzed using Spectronaut with default settings.

## HE staining and pathomic preparation

The large size of the WSI prevents direct extraction of features using deep learning network. We therefore used a patch-based deep learning feature extraction to scan and label the WSI region of interest in the marked primary Gleason patterns, which was consistent with the proteomics samples preparation. The open slide library was then used to divide the non-overlapping slices of the region of interest of WSI into 256*256-pixel patches. To reduce inconsistency in dyeing differences, we adopted dyeing normalisation (colour normalisation) technology. The staining of the cut patches was normalised using the Macenko technique [26]. We selected ResNet50 [27], a widely used convolutional neural network with established model capability, as our feature extraction model to extract deep learning features from WSI. The ResNet50 network consists of 49 convolutional layers and a fully connected layer that serves as a classifier. During the feature extraction, we loaded the parameters of the first 49 convolutional layers of the ResNet50 pre-trained model. Only the last fully connected layer was altered to obtain 2048 output dimensions. After the features of each patch were obtained, the deep learning features of all patches in the same WSI were averaged to obtain the 2048-dimensional deep learning features of the whole WSI.

## MRI acquisition and radiomic feature extraction

Radiomic feature extraction plays a crucial role in radiomic analysis, facilitating the identification of disease-relevant imaging features from extensive image datasets. These features encompass various morphological characteristics, including tumour size, shape, and contour, as well as texture density. This provides valuable support for medical, research, and clinical applications.

The workflow, presented in Figs. 5A and 6A, illustrates the overall process. Among the currently available methods of segmentation, manual segmentation performed by experts is recognized as the most accurate “gold standard”. In our study, a radiologist conducted the image segmentation. The region of interest (ROI) for the prostate in all patients was selected using 3D Slicer (version 4.8.0; http://www.slicer.org). Image pre-processing and feature extraction were conducted using the open-source Pyradiomics package (http://www.radiomics.io/pyradiomics.html). This package supports multiple image data formats and offers extensive options for feature calculation and flexible parameter adjustment, simplifying and enhancing the efficiency of the feature extraction and analysis. Re-sampling was performed utilising the BSpline interpolator in the SimpleITK package, known for its ability to preserve fine details in the image. A standard voxel spacing of 2 × 2 × 2 mm was used. The voxel intensity values were discretised with a bin width of 25 HU to reduce image noise and normalise the intensities.

The extracted radiomic features were organised into different categories, including First Order, Shape, Gray-Level Co-occurrence Matrix (GLCM), Gray-Level Size Zone Matrix (GLSZM), Gray-Level Run Length Matrix (GLRLM), Neighbouring Gray Tone Difference Matrix (NGTDM), and Gray-Level Dependence Matrix (GLDM) features. Within each three-dimensional segmentation, the radiomic features were grouped as follows: shape (*n* = 26), first-order (*n* = 324), GLCM (*n* = 397), GLSZM (*n* = 288), GLRLM (*n* = 288), NGTDM (*n* = 25) and GLDM (*n* = 252). Ultimately, a total of 1600 features were obtained from each VOI per sequence and per patient.

## Feature screening and validation based on machine learning

**Fig. 1:**
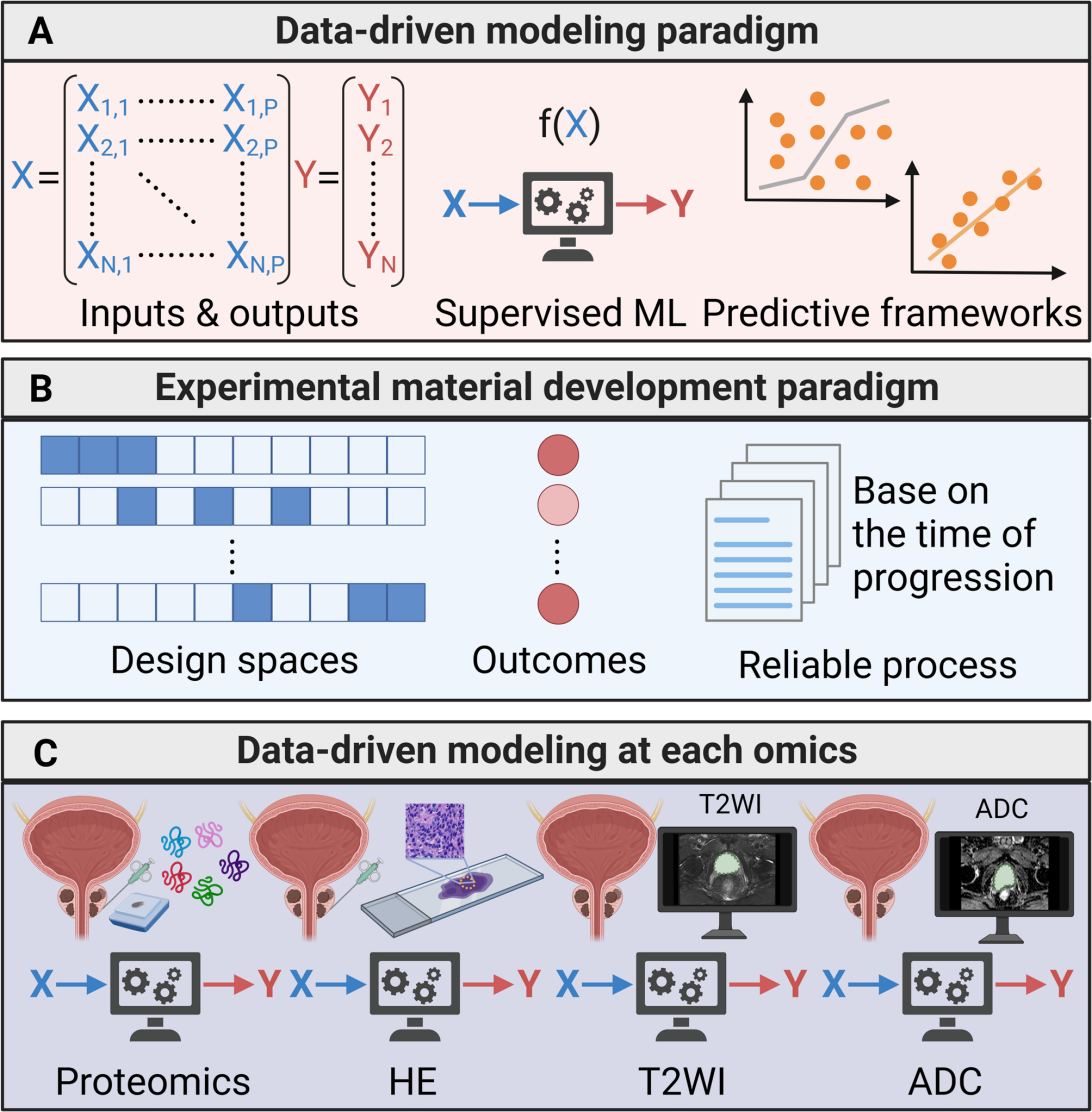
An Integrated Multi-Omics Data-Driven Experimental Study of HSPC. (**A**) Data-Driven Modelling (DDM) Paradigm A supervised machine learning approach is employed to predict HSPC progression within the data-driven modelling framework. (**B**) Experimental Paradigm The experimental paradigm utilizes multi-omics to optimize the recognition of HSPC progression. (**C**) Modular Approach Integration Our modular approach integrates DDM with experimentation to map input-output relationships at each stage of soft granular matrix development. Additionally, it integrates DDM with each omics model to map input-output relationships in the multi-omics study of HSPC

Detailed steps of machine learning in this paper are shown in Fig. 1. The mass spectrometry proteomics data are available from the iProX with the dataset identifier IPX0005031000 [20]. In order to identify the features related to survival time under each mode, we used the Boruta algorithm for feature screening. In contrast to previous algorithms designed to select the feature that minimises the loss function in the current model, the Boruta algorithm is intended to screen out all the feature sets that are correlated with the outcome variable [28]. The Boruta algorithm scores the importance of the current feature by constructing a random number for each feature as a substitute. If the effect is better than the random feature during training, the feature is deemed useful and significant. Thus, the algorithm ranks the importance score. The average value of feature importance is obtained after iteration. We controlled for the stringency of feature screening, and for each data mode we screened approximately 60 features, which were most relevant to survival time.

**Fig. 2:**
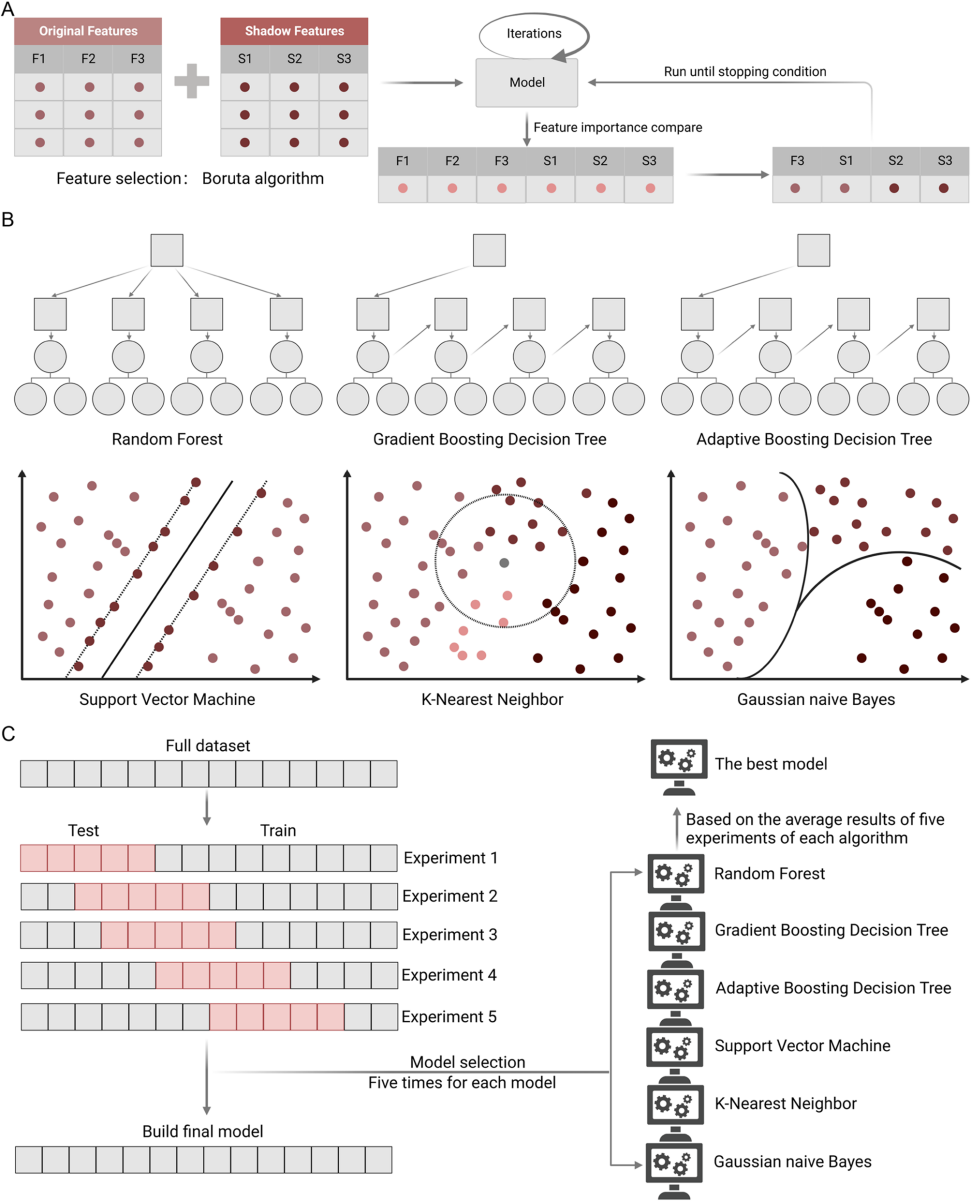
Machine Learning Algorithms and Training Process. (**A**) Boruta Algorithm.The Boruta algorithm generates feature importance scores in each iteration, with key features iteratively added to the original feature set until stopping criteria are met.(**B**) Base Algorithms.Base algorithms—including Random Forest, Gradient Boosting Decision Tree, Adaptive Boosting Decision Tree, SVM, K-Nearest Neighbor, and Gaussian Naive Bayes—were tuned and selected for each omics study. (**C**) Model Evaluation Workflow.The modeling workflow was evaluated via five independent experiments for each model

In this study, the machine learning - related results were obtained using the scikit - learn package in the Python 3.12 environment. Specifically, the following classifiers were employed for analysis: “GradientBoostingClassifier”, “RandomForestClassifier”, “AdaBoostClassifier”, “SVC”, “KNeighborsClassifier” and “GaussianNB”. Then through five independent repeated experiments, randomly divided the patients into training and testing sets in a ratio of 7:3 for each experiment, we used six machine learning methods (Gradient Boosting Decision Tree (GBDT) [29], Random Forest (RF) [30], AdaBoost [31], SVM [32], K-Nearest Neighbor, Gaussian Naive Bayes) to verify the classification performance of the features screened under each mode. Figure 2 presents the model diagram. The results of multi-omics computed by six machine learning methods are summarized in Table 2.

**Table 2:** Detailed results of multi-omics in six machine learning

	GBDT	Random Forest	AdaBoost	SVM	K-Nearest Neighbor	Gaussian naive Bayes
*Proteomics*						
auc	0.6284	0.8522	0.7861	0.8928571	0.7707	0.7051
acc	0.625	0.85	0.7917	0.875	0.7583	0.7
Recall	0.6705	0.9159	0.8064	1	0.8543	0.7712
Precision	0.6148	0.7997	0.8183	0.7857143	0.6797	0.6454
*Pathomics*						
auc	0.6662	0.9583	0.7522	0.9655	0.8588	0.9398
acc	0.675	0.9583	0.7583	0.9667	0.85	0.9417
Recall	0.7144	0.9231	0.7517	0.9399	0.9485	0.8989
Precision	0.6444	1	0.811	1	0.7723	1
*The DWI-ADC MRI module of radiomics*						
auc	0.6281	0.6671	0.8461538	0.5546	0.6101	0.551
acc	0.6	0.6333	0.8333333	0.525	0.6	0.525
Recall	0.7193	0.7732	1	0.6118	0.6598	0.5703
Precision	0.5203	0.5553	0.6923077	0.6145	0.6187	0.632
*The T2 WI MRI module of radiomics*						
auc	0.7083	0.8461538	0.6138	0.4846	0.674	0.6351
acc	0.6917	0.8333333	0.6	0.425	0.6417	0.6
Recall	0.8064	1	0.6679	0.4861	0.7755	0.7965
Precision	0.5949	0.6923077	0.5794	0.5692	0.6052	0.3967

GBDT, RF, and AdaBoost belong to ensemble learning. The two common ensemble learning frameworks include Bagging and Boosting. Bagging employs parallel ensemble training for the model during the training, while Boosting uses serial ensemble training. GBDT is a well-known algorithm with enhanced generalisation ability and represents the Boosting strategy. Each weak classifier depends on the result of the previous weak classifier for model optimisation based on the fastest gradient descent. The RF algorithm is an extended variant of the Bagging strategy algorithm. Samples and features are randomly selected to form decision trees. Decision trees are independent of each other. Evaluation under each decision tree is independent, and the results of classification are based on the voting method. AdaBoost also belongs to the Boosting strategy, which adaptively enhances the misclassification of the previous basic classifier. The weighted whole sample is used to train the next basic classifier.

In addition to the three commonly used integration algorithms mentioned above, we also utilised three widely used algorithms to verify the extracted features. The SVM is primarily designed to resolve the separate hyperplane that can correctly partition the training data set and has the largest geometric spacing. It is a widely adopted algorithm for classification tasks. The K-Nearest Neighbor determines the nearest neighbour based on the closest one or several samples. Despite its simplicity, the K-nearest neighbor is better than complex algorithms in some tasks. Gaussian Naive Bayes assumes that the conditional probability of each feature dimension in the data set conforms to the Gaussian distribution. Compared with other naive Bayes classification models, the Gaussian Naive Bayes classification model is more consistent with natural data distribution and is more widely used for Recall and Precision as indicators to assess the effectiveness of classification. In this study, the LASSO logistic regression algorithm was used to address feature selection in high-dimensional multi-omics data, with penalty parameter tuning via 10-fold cross-validation that partitioned the dataset into 10 subsets for training and validation to optimize the penalty parameter and minimize prediction error [33]. Using the R “glmnet” package, we integrated progression time, disease status, and multi-modal feature expression data, then applied the lasso-Cox method for survival-based regression analysis to further screen and optimize features [34]. This two-stage approach—initial screening via LASSO logistic regression for binary outcomes and subsequent lasso-Cox modeling for time-to-event data—systematically reduced the initially selected 45 features to 30, ensuring the final set balanced discriminative power for disease status, prognostic value for progression, and resistance to collinearity and overfitting.

### Development and validation of a multi-omics-based model

In the last phase, the multi-variable Cox regression (R package “survival”) was used to determine the prognostic significance of 30 features derived from the LASSO model: In addition, the R package “forestplot” was used to visualise each variable (p-value, hazard ratio (HR), and 95% confidence interval (CI)).

A nomogram is a visual tool constructed based on multivariate statistical models, designed to integrate multi-dimensional predictors for estimating individual outcome probabilities (e.g., disease risk, survival rate). Next, the nomograms were created using the “RMS” software to predict the disease progression rates after 12, 18, and 24 months. The calibration curve showed the performance of the nomograms with the rates observed at 12, 18, and 24 months. Finally, we obtained the area under the curve (AUC) via receiver operating characteristic (ROC) analysis using the R package “pROC”. In particular, we determined the patients’ follow-up durations and risk scores, and performed the ROC analyses at 12, 18, and 24 months. In nomograms, the cutoff value serves as a critical threshold for classifying individual predicted probabilities into different risk tiers, and its interpretation requires integrating statistical evidence, clinical needs, and model validation results. Specifically, cutoff values are typically determined based on the maximum Youden’s index from receiver operating characteristic (ROC) curves or the maximization of net benefit in decision curve analysis (DCA).

## Results

### Proteomics feature selection and stratification

**Fig. 3:**
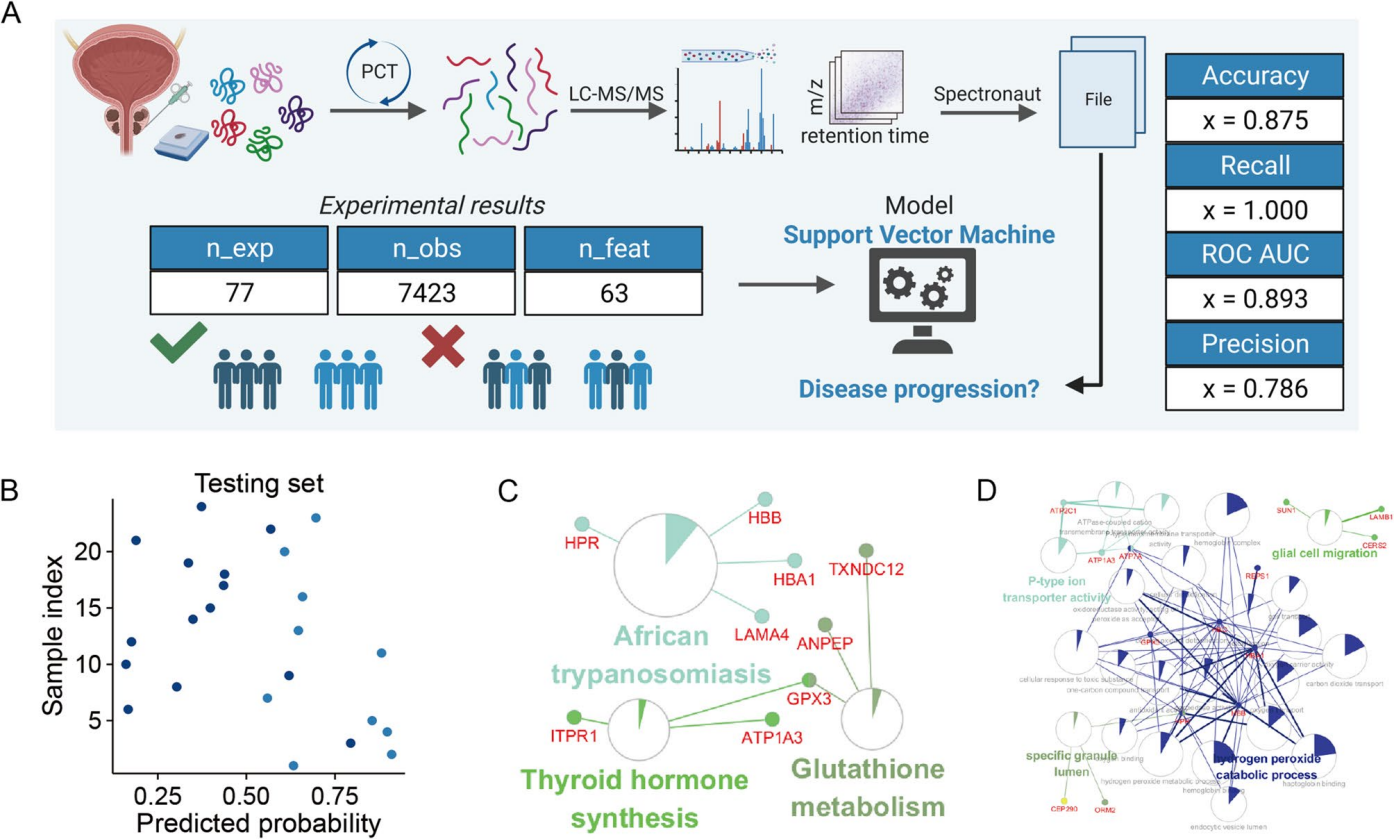
Feature Extraction and Functional Analysis of Proteomics**. ** (**A**) Overview of PCT-Assisted Proteomics Workflow and Data-Driven Models. Summary boxes depict the number of unique experiments, recorded observations, and input design parameters in the PCT-assisted proteomics fabrication and corresponding data-driven modeling pipeline. (**B**) Prediction Probability of the Optimal Model on the Test Set. Dark blue dots represent HSPC with long-term progression, while light blue dots represent HSPC with short-term progression, illustrating the model’s predictive performance. (**C**) KEGG Pathway Analysis of Protein Features. (**D**) Gene Ontology (GO) Analysis of Protein Features

Using proteomic data including 7423 protein expression, we screened 63 features related to the patients prognosis. We found that SVM was the most appropriate machine learning method for proteomic feature selection to distinguish the risk of rapid CRPC progression, and the AUC was 0.893 (Fig. 3A). We further performed KEGG and GO analysis of these 63 screened features. The KEGG pathway was mainly expressed in African trypanosomiasis (KEGG:05143), thyroid hormone synthesis (KEGG:04918), and glutathione metabolism (KEGG:00480). GO was mainly enriched in P-type ion transporter activity (GO:1990748), specific granule lumen (GO:0035580), hydrogen peroxide catabolic process (GO:0042744), and glial cell migration (GO:0008347).

### Histopathological feature selection and stratification

**Fig. 4:**
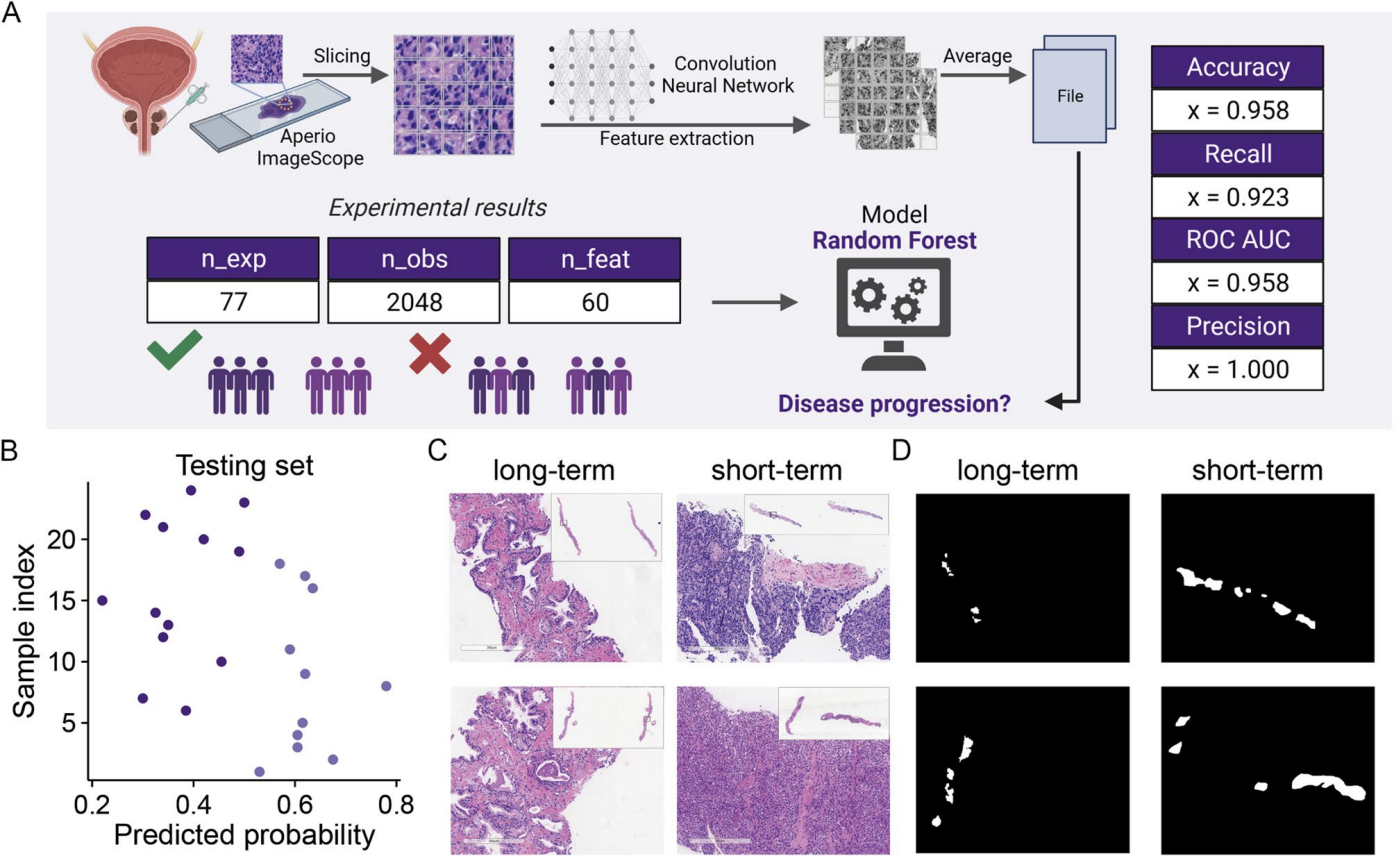
Construction of Digital Pathomics. (**A**) Overview of Pathomics Analysis for H&E Staining Slides via Deep Learning. Summary boxes display the number of unique experiments, recorded observations, and input design parameters in the pathomics analysis workflow for constructing H&E staining slides using deep learning. (**B**) Prediction Probability of the Best Model on the Test Set. Differently colored dots represent the true labels of test samples in the prediction probability plot of the best model based on the test set. (**C**) Partial Views of WSIs at Different Progression Times. (**D**) Regions of Interest Masks at Different Progression Times

We labelled primary Gleason patterns on 77 H&E WSIs, generating tens of thousands of tiles, each measuring 256 × 256 pixels (Fig. 4a). The ResNet-50 convolutional neural network pre-trained on ImageNet extracted the 2048-dimensional patch-based deep learning features. Based on the 60 features selected by the Boruta algorithm, the risk of patients with rapid CRPC progression were distinguished with an accuracy of 0.958. Results are shown in Fig. 4.

### MRI feature selection and stratification

Feature extraction is an essential step in radiomic analysis. It helps us identify imaging features relevant to diseases from a massive amount of image data. These features include morphological characteristics such as tumour size, shape, and contour, as well as textural features such as texture density. These features facilitate medical, research, and clinical applications. Manual segmentation performed by experts is considered the most accurate “gold standard.” In this study, the lesion on MRI was manually delineated layer by layer by physicians using 3DSlicer, resulting in the generation of a 3D volume of interest (VOI). After segmentation, we performed radiomic feature extraction on the MRI using the open-source Python package Pyradiomics. This package supports various image data formats and provides options for rich feature calculation and flexible parameter adjustment, thereby simplifying and enhancing feature extraction and analysis. The extracted radiomics features were stratified into First Order, Shape, GLCM, GLSZM, GLRLM, NGTDM, and GLDM types. The radiomics features based on each three-dimensional segmentation were classified as follows: (1) shape (*n* = 26), first-order (*n* = 324), GLCM (*n* = 397), GLSZM (*n* = 288), GLRLM (*n* = 288), and GLDM (*n* = 252).

In radiomics, GLCM (Gray-Level Co-occurrence Matrix), GLSZM (Gray-Level Size Zone Matrix), GLRLM (Gray-Level Run Length Matrix), NGTDM (Neighborhood Gray-Tone Difference Matrix), and GLDM (Gray-Level Difference Matrix) are all texture analysis tools based on pixel intensity distribution. Their clinical relevance lies in assisting disease diagnosis, prognostic assessment, and treatment response prediction by quantifying image texture features. For example, GLCM can differentiate benign and malignant tumors, GLSZM evaluates the complexity of the tumor microenvironment, GLRLM reflects tumor differentiation degree, NGTDM indicates tissue heterogeneity, and GLDM aids in predicting treatment response. These features provide supplementary information for clinical decisions by capturing subtle structural differences undetectable by the naked eye in conventional imaging, holding significant value in precision tumor subtyping and personalized therapy. Ultimately, 1575 features were obtained from each VOI per sequence per patient. Features were obtained via wavelet filtering processing. The mathematical meanings of these radiomic signatures have been described previously [35] and are available at https://pyradiomics.readthedocs.io/en/latest/.

**Fig.5:**
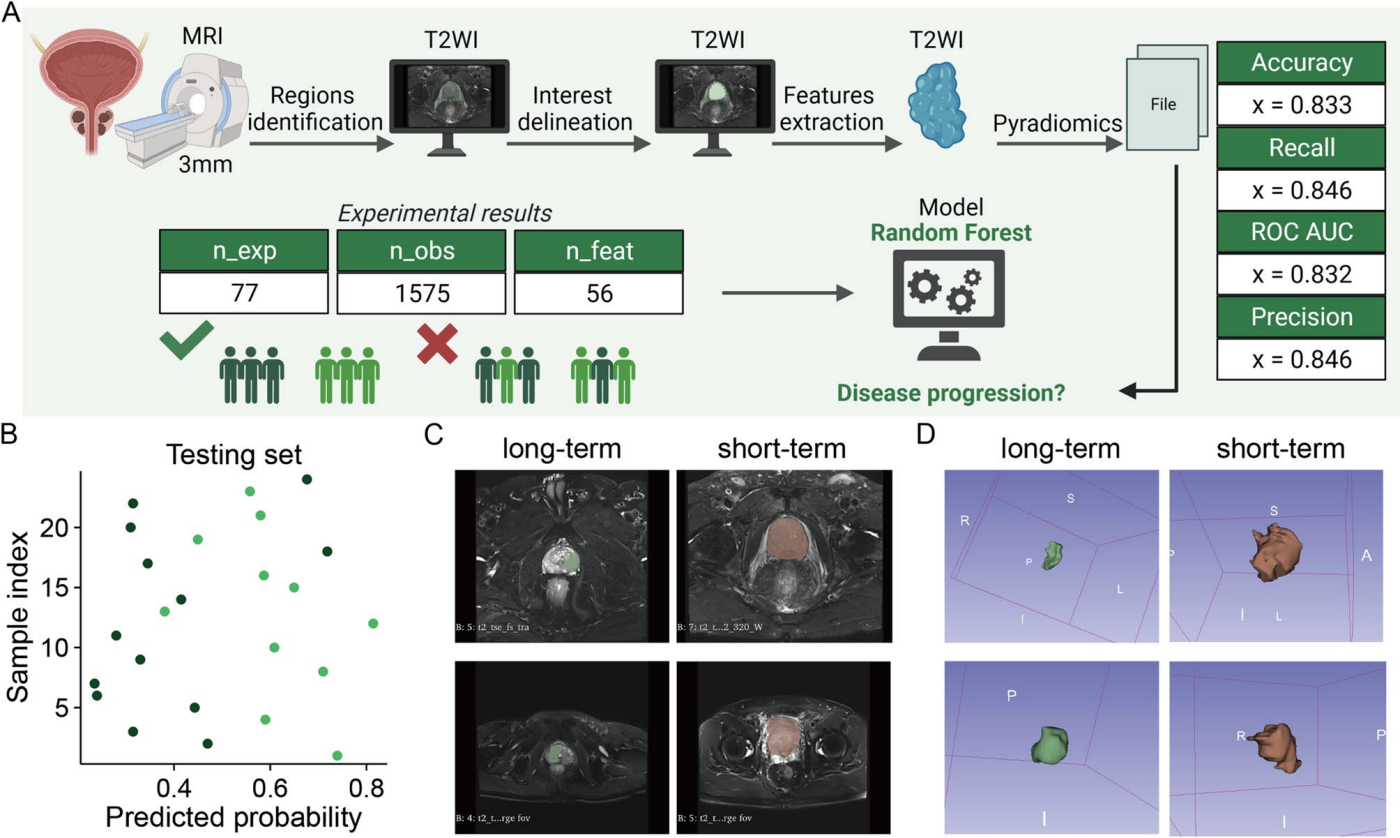
Construction of the DWI-ADC MRI Module in Radiomics. (**A**) MRI DWI-ADC sequences were used for HPSC region identification and delineation of regions of interest (ROIs), followed by feature extraction using 3D Slicer to obtain radiomic signatures. (**B**) Prediction probability of the optimal model on the test set, where differently colored dots represent the true labels of test samples. (**C**) MRI DWI-ADC sequences at varying progression time points. (**D**) Manually segmented tumours at different progression time points

**Fig. 6:**
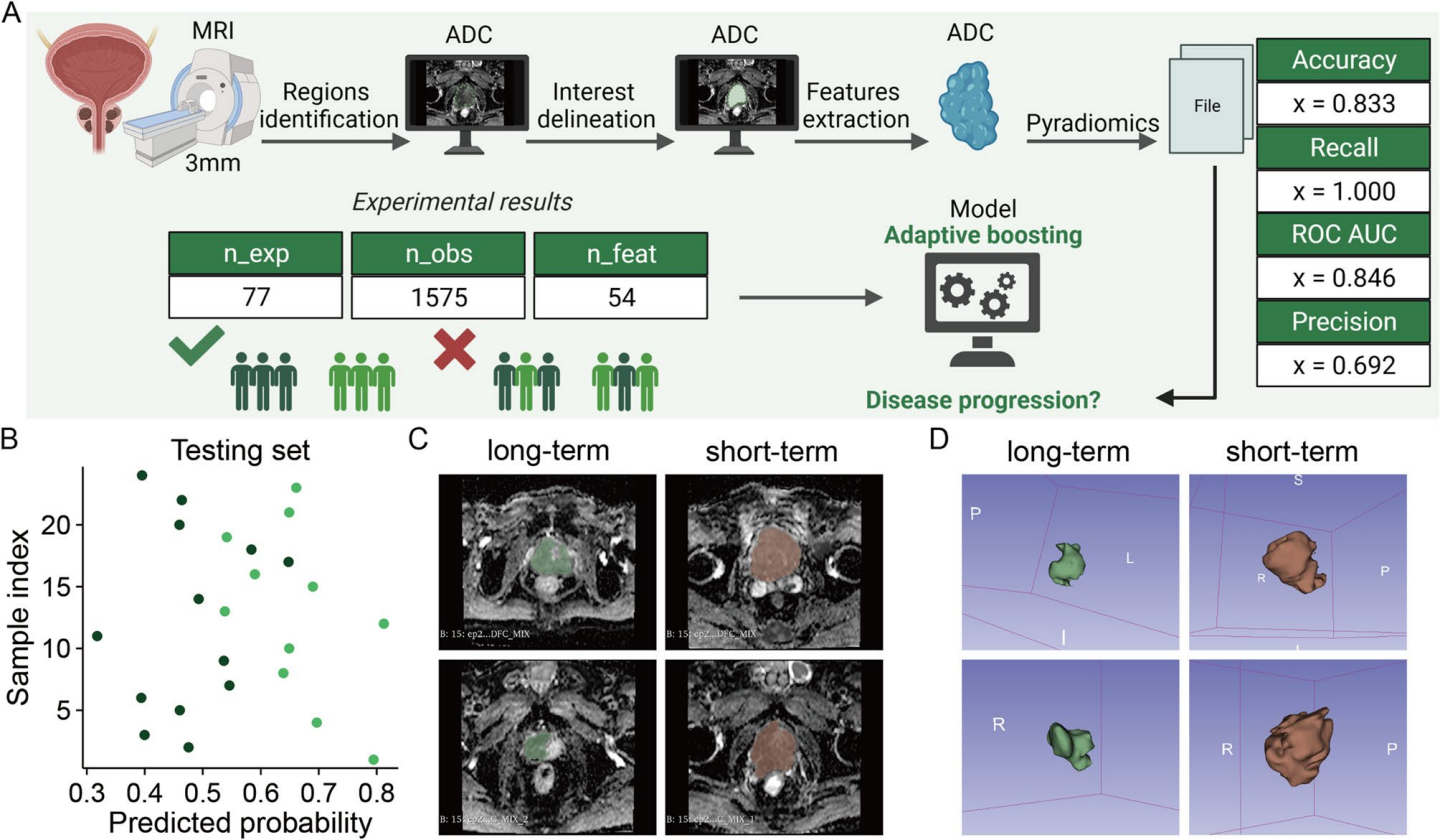
Construction of the T2 WI MRI Module in Radiomics.(**A**) MRI T2 WI sequences were used for HPSC region identification and delineation of regions of interest (ROIs), followed by feature extraction using 3D Slicer to obtain radiomic signatures. (**B**) Prediction probability of the best model on the test set, where differently colored dots represent the true labels of test samples. (**C**) MRI T2 WI sequences at different progression time points. (**D**) Manually segmented tumours at various progression time points

In this sequence, we selected the 3-mm VOI subgroup, which is the most commonly used in clinical practice. Next, two radiomic models based on VOI 3 mm of T2 WI and DWI-ADC sequences exhibited ideal and stable predictive efficacy (AUCs = 0.833). Nonetheless, the RF was most suitable for MRI-T2 WI and AdaBoost was most appropriate for MRI-ADC (AUCs = 0.846). Results are shown in Figs. 5 and 6

### Multi-modal prognostication

**Fig. 7:**
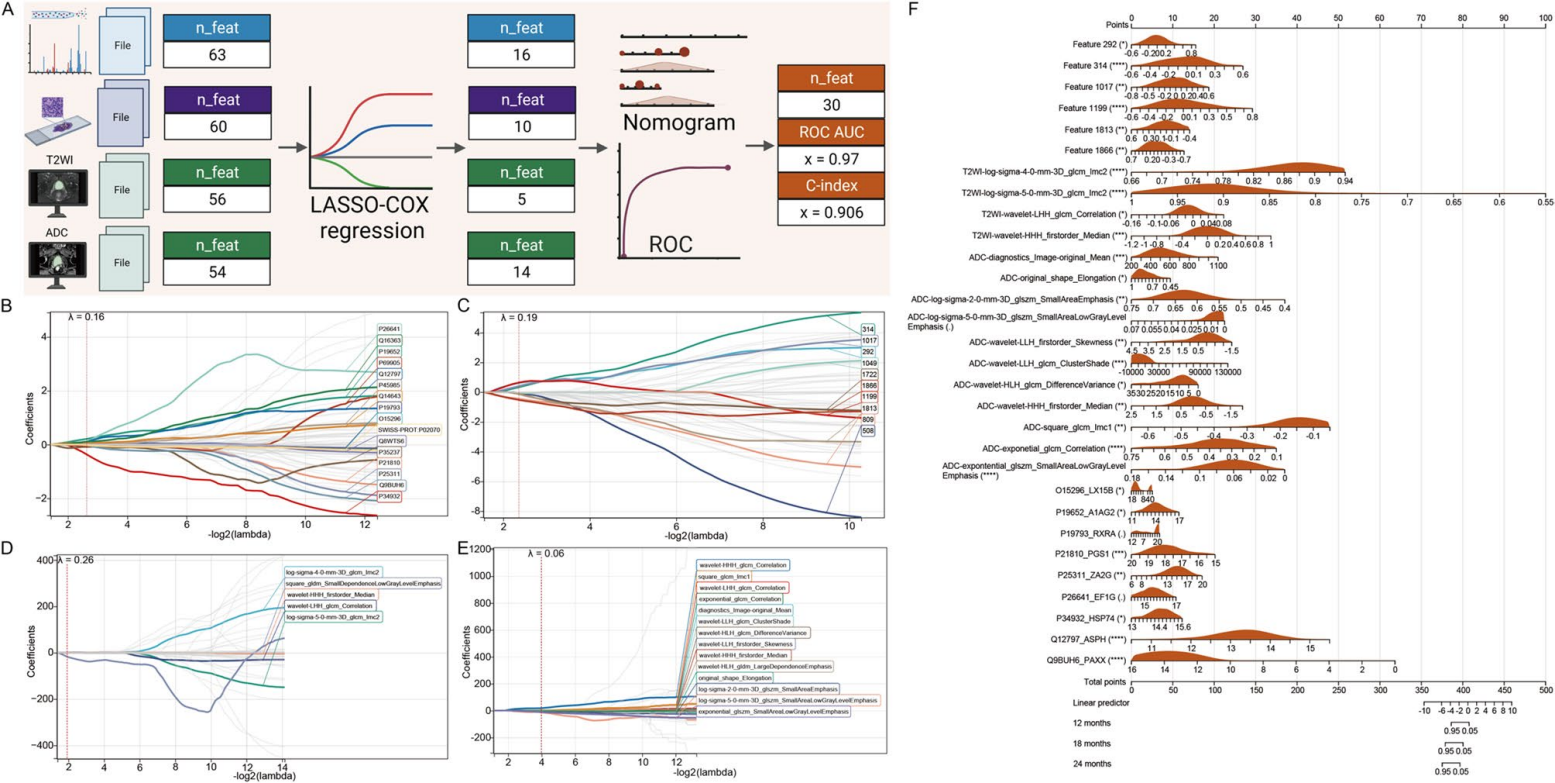
Construction of a multi-omics model. (**A**) Overview of Multi-Omics Data Screening and Model Construction. The workflow depicts the process of multi-omics data screening and subsequent model construction. (**B**-**E**) LASSO Coefficient Profile Plots with Varying log(λ)LASSO coefficient profile plots are shown for (**B**) proteomics (λ=0.16), (**C**) patholomics (λ=0.19), (**D**) T2 WI radiomics (λ=0.26), and (**E**) DWI-ADC radiomics (λ=0.06), each corresponding to different log(λ) values. (**F**) Construction of a Nomogram for the Multi-Omics Model

Overfitting is likely due to multiple predictors in the classification model. We thus built a LASSO regression model. LASSO is a regularisation technique that can be used to reduce the magnitude of coefficients and avoid overfitting. In addition to the features extracted from proteomics, histopathology and pyradiomics, we conducted a LASSO regression analysis. We screened 16 proteomics features, 10 histopathological features, 5 MRI-T2 WI features and 14 MRI-ADC features. Finally, we implemented a fusion approach to integrate proteomics, histopathology, and pyradiomics into a complex model. Specifically, we used multi-factor COX regression to further screen features and use the nomogram as a visual evaluation tool, which finally incorporated 30 multi-omics features. The AUC of this nomogram model was 0.97 and the C-index was 0.906. The accuracy of the fusion model is markedly higher than that of any single model, which greatly improves disease recognition. Results are shown in Fig. 7.

## Discussion

In this study, we aimed to identify the patients at high risk of rapid progression from hormone-sensitive prostate cancer (HSPC) to lethal castration-resistant prostate cancer (CRPC). We previously reported that multi-modal approach may play a key role in the rapid progression of HSPC to CRPC. This work has highlighted important prognostic insights based on proteomics data, pyradiomics and histopathological specimens, which were analysed via machine learning. Finally, we have developed a multi-omics-based nomogram model that can be used to identify HSPC patients with high risk of rapid progression and predict the progression free survival.

Clinical staging of newly diagnosed patients with PCa in China differs from that of countries in the West. For instance, most of the patients (76%) with newly diagnosed PCa in the United States are clinically localized, while only ~ 13% and ~ 6% involve metastases involving local lymph nodes or distant sites, respectively. However, the Chinese data differ significantly. A multi-centre Chinese study showed that only a third of the newly diagnosed PCa patients are clinically localised. Further, most patients in China are in the middle or advanced stage of PCa diagnosis, resulting in a worse overall prognosis than in Western countries. For this reason, our study focused on investigating advanced PCa cases from China. In our study, we enrolled 77 patients with advanced PCa, but their median time for progressing to CRPC was only 9 months: substantially shorter than those reported previously [36]. This fact may be explained by the presence of metastases in most of our enrolled patients (*N* = 70).

Recent studies have shown that the time of HSPC progression to CRPC is highly variable in patients treated with standard ADT [37]. Multiple phase-III trials proved the importance of identifying patients with HSPC at risk of rapid disease progression, for prompt intervention with appropriate therapeutic strategies to improve the prognosis [38]. Metastatic HSPC risk classification has been reported in two studies. In the first study known as the CHAARTED trial, the patients with visceral and/or at least four bone metastases were classified as high-volume and distinguished from the remaining low-volume ones [39]. The high-volume group benefited from ADT + docetaxel treatment, whereas the low-volume group was treated with ADT alone. The second study known as the LATITUDE trial included patients with at least two high-risk characteristics (at least three bone metastases, visceral metastases, and ISUP grade 4), and reported increased survival following abiraterone acetate plus prednisone therapy [40]. However, several patients defined as low-risk or low-volume progressed to CRPC rapidly in the LATITUDE trial. In our study, most patients would have been classified as low-volume and low-risk according to the CHAARTED and LATITUDE criteria, respectively. However, our patients exhibited rapid disease progression (median time: 9 months). Collectively, stratifying patients based on clinical imaging or M stage alone may be inappropriate. With the gradual application of artificial intelligence and machine learning techniques in clinical practice, new methods can be used for disease diagnosis, prediction, and even formulation of treatment plans [41].

Advances in quantitative biomarker development have provided new forms of data-driven insights into patients with cancer. However, most approaches are based on a single mode for data acquisition, while approaches integrating different modalities remain relatively underdeveloped. Multi-modal integration of advanced molecular diagnostics, radiological, and histological imaging strategies, and codified clinical data presents opportunities for advanced precision oncology beyond genomics and standard molecular techniques [25]. The central premise of multi-modal data integration is that orthogonally-derived data complement one another, thereby augmenting information content beyond that of any individual modality. Each modality is incomplete and often noisy, but integrating weak signals across modalities can overcome noise associated with a single modality and more accurately infer response variables of interest. Based on this perspective, we analysed complementary modalities of data analysis combined with emerging multi-modal artificial intelligence methods. Such a strategy can lead to the emergence of a re-imagined class of multi-modal biomarkers to propel the field of precision oncology in the coming decade. Models with improved risk stratification developed herein can be used to extract and integrate artificial intelligence-based quantitative clinical multi-modal features to facilitate urologists in selecting primary treatment, planning surveillance frequency, making decisions about maintenance therapy, and counselling patients regarding clinical trials of investigative agents. The statistical robustness and clinical relevance of the risk groups based on disease progression in the test set underscore the utility of multi-modal machine-learning approach, establishing a proof of principle. Nonetheless, the results of different machine learning models differ based on feature data. Overall, the three methods of integrated learning perform well. GBDT was effective based on MRI features, but yielded average results based on protein and WSI features. Regardless of the characteristics, both RF and AdaBoost were moderate to excellent in terms of performance, among the six models reviewed. SVM performed best for features derived from deep learning, such as WSI features; however, it was mediocre based on features containing specific information, such as MRI image omics. The performance of K-Nearest Neighbor was moderate in every feature, suggesting that it is always an option as a stable algorithm that adapts to any feature. Gaussian naive Bayes showed excellent performance based on WSI features and average performance in the presence of other features. No machine learning model can always outperform others to achieve the best performance for different tasks and features. When encountering different tasks, the machine learning algorithm that is most suitable for the current data should be selected based on the characteristics of the task.

To date, only a single HSPC proteomic data has been reported. This study cited our previous proteomic data and further optimising the algorithm to show better recognition (AUC = 0.893). This study identified 9 proteins valuable for HSPC classification. Among them, Aspartate Beta-Hydroxylase (ASPH) and PAXX Non-Homologous End Joining Factor (PAXX) are the most significant. ASPH is mostly correlated with the CRPC phenotype [42]. In CRPC cells, the inhibition of ASPH expression mediated via specific small interfering RNA or culturing cells under hypoxic conditions, reduced cell proliferation, invasion and cyclin D1 expression. The inhibition was achieved via modulation of NOTCH signaling. PAXX is one of the key proteins of the Non-homologous DNA End Joining (NHEJ) involved in the DNA double-strand break (DSB) repair pathway [43]. The association between PAXX and prostate cancer has not been reported. Meanwhile, the pathway analysis of differential proteins based on these findings and previous studies suggest that HSPC progression is closely related to glutathione metabolism [44].

In general, multi-modal integration with histopathological imaging improves outcome predictions and stratification compared with uni-modal and molecular methods alone [45–47]. Pathology specimens depicting cell morphology, tissue architecture, and tumour–immune interfaces are increasingly digitised [48]. Currently, histopathological deep learning analysis of prostate cancer is based on molecular classification [49], tumour grading [50], and personalised treatment [51]. Gleason score still remains the pathologic standard for evaluation of prostate cancer. Clinical data have confirmed that the Gleason classification is not accurate enough to evaluate patients’ prognosis. However, currently, no histopathological deep learning technique is available for HSPC. For histopathological imaging, we developed an H&E WSI-based model to stratify HSPC patients. Recent evidence shows that small models without pre-training, such as ResNet-50, for small medical imaging datasets are comparable to pre-trained large models [52]. Therefore, we adopted ResNet-50 for the feature extraction in our model. The results suggest that the differentiation accuracy for patients was as high as 0.958.

Routine segmentation of the whole tumour volume is impractical in daily practice using current tools due to the prohibitively high demand for time and expertise. Radiomics is an emerging tool for image analysis. It can be used to extract multiple quantitative features from imaging data to quantify tumour heterogeneity, which is significant for personalised oncology [53]. We used T2 WI and ADC sequences to extract features. T2 WI can reveal the tumour anatomy as well as perineural and seminal gland involvement in prostate cancer. Further, the images exhibit valuable textural features. ADC values objectively reflect the degree of diffusion of water molecules in biological tissue. They correlate with tumour malignancy, avoiding the penetration effect of DWI due to the very long T2 decay time of the tissue. The combination of T2 WI and ADC provides accurate and comprehensive tumour information. TWI-log-sigma-4-0-mm-3D_GLCM_IMC2 and TWI-log-sigma-5-0-mm-3D_GLCM_IMC2 were the most significant in MRI-T2 WI, suggesting that shape is crucial in distinguishing HSPC in T2 WI. In MRI-ADC, ADC-Exponential_GLCM_Correlation and ADC-Exponential_giscrcration functions suggest a small area with low gray level and the most significance. Therefore, GLCM is the key to distinguishing HSPC among ADC. Most previous studies also demonstrated the importance of texture based on GLCM of MRIs as a pathological indicator in PCa [54–56].

Machine learning in cancer prognostics is a growing field with significant potential. However, the contribution of common diagnostic modes to multi-modal risk stratification remains poorly understood. Here, we show that our integrating multi-omics model could help identify patients at high risk of rapid CRPC progression, and aid clinicians in predicting the progression free survival of patients, guiding personalized clinical management and decision-making. Our fusion architecture benefits from fewer parameters, which reduces overfitting. One drawback of this study is the limited sample size. The absence of extensive proteomics data or clinical follow-up contributed to the small sample size. Finally, nomograms also have limitations, primarily including strong dependence on high-quality multi-dimensional clinical data (often affected by data missing, noise, and insufficient standardization), high implementation costs (some predictors require complex testing or professional operation), inadequate dynamic adaptability (difficult to adapt to heterogeneous populations or incorporate new biomarkers), delayed clinical validation (mostly lacking external independent validation), the black-box effect (complex model logic affecting trust in results), and standardization challenges (process variations across institutions prone to causing biases). Their clinical translation requires technological simplification, multi-center validation, and interdisciplinary collaboration.In the future, large multi-centre trials with increased numbers of clinical cases are needed to ensure the universality of the study.

## Data Availability

The mass spectrometry proteomics data are available from the iProX with the dataset identifier IPX0005031000. All the data analyzed during the current study are available from the corresponding author on reasonable request.
